# Lobular Capillary Hemangioma of Middle Turbinate Literature Survey and Case Report

**Published:** 2012

**Authors:** Mohammad Reza Majidi, Amir Hossein Jafarian, Ayeh Shahabi

**Affiliations:** 1 *Ear, Nose and Throat Research Center, Ghaem Hospital, Faculty of Medicine, Mashhad University of Medical Sciences, Mashhad, Iran*; 2 *Department of pathology Ghaem Hospital Faculty of Medicine, Mashhad University of Medical Sciences,Mashhad, Iran*; 3 *Department of otorhinolaryngology, Ghaem Hospital, Faculty of Medicine, Mashhad University of MedicalSciences, Mashhad, Iran*

**Keywords:** Lobular capillary hemangioma, Nasal cavity, Turbinate

## Abstract

**Introduction:**

Lobular capillary hemangioma (LCH) is a benign lesion of vascular origin. It rarely involves nasal cavity which most commonly manifests as progressive nasal obstruction and epistaxis.

**Case Report:**

In this report we present a case of LCH of the nasal cavity which occurred approximately one month after delivery. There was no recurrence after complete endoscopic resection during one year follow up.

## Introduction

Lobular capillary hemangioma (LCH) is a benign lesion of vascular origin. The most common sites of lesion are the skin and oral mucosa. LCH rarely involves nasal cavity which most commonly manifests as progressive nasal obstruction and epistaxis. Trauma and hormonal changes are presumed as two major predisposing factors (1-4). In this report, we present a case of LCH of the right middle turbinate. 

## Case Report

A 32-year-old woman came to hospital clinic with complaint of progressive right-sided nasal obstruction associated with intermittent mild epistaxis, muco-purulant rhinorrhea and post nasal drip since 10 months ago began one month after her uncomplicated delivery of a normal baby. She was completely asymptomatic during her pregnancy.. She didn’t mention any history of nasal trauma or nasal intubation but she has had history of multiple nasal packing during recent months. Endoscopic examination of nasal cavity revealed a red large polypoid mass which bled easily and originated from posterior part of middle turbinate.

**Fig 1:**
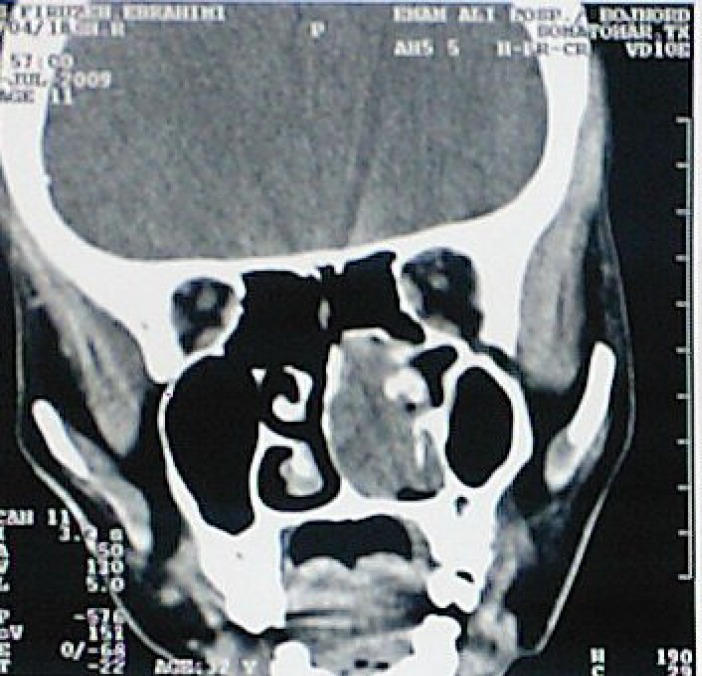
Paranasal sinus CT scan shows Intranasal mass lesion

A CT scan of the paranasal sinuses also revealed a right nasal cavity soft tissue mass without any evidence of bony destruction or extension of the mass into the adjacent paranasal sinuses (fig 1).

**Fig 2:**
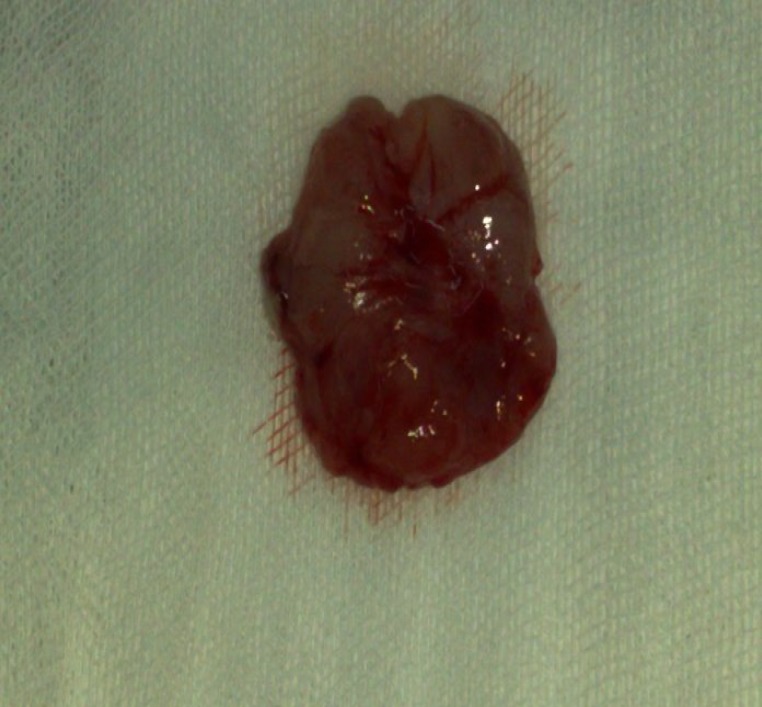
Gross appearance of the nasal mass after excision

The patient was taken to the operating room where the soft tissue mass was completely excised using endoscopic techniques. The surgery involved partial resection of the middle turbinate (Fig 2).

**Fig 3:**
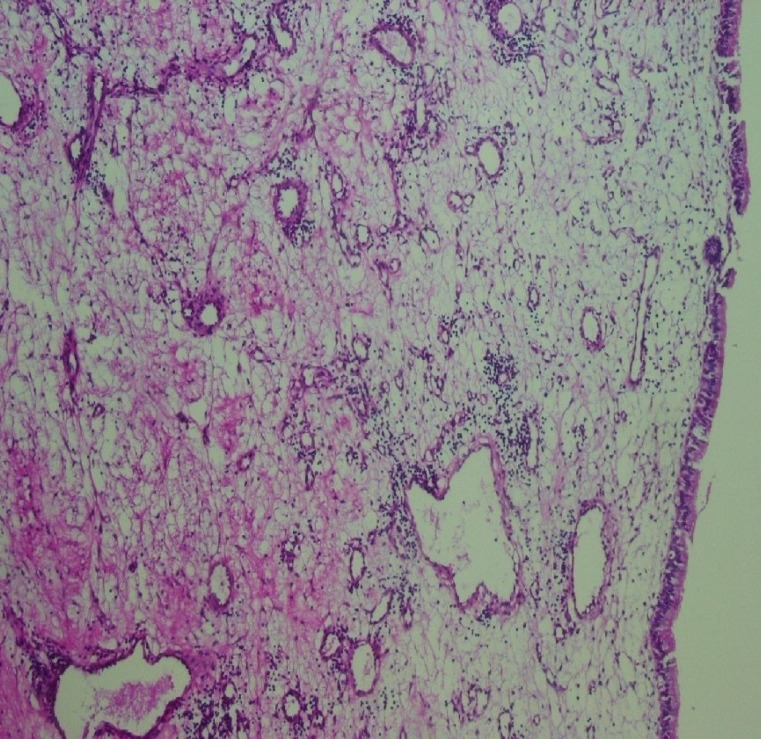
Loboular capillary hemangioma composed of small capillaries lined by plump endothelial cells (H&E staining ×100)

Histologic finding showed proliferation of small capillaries lined by plump endothelial cells in a loboular pattern (Fig 3).

There was no recurrence during the one-year follow-up period.

## Discussion

Hemangiomas are vascular neoplasms that morphologically classified in to capillary, cavernous, arteriovenouse and epitheliod type (5). LCH, which has previously been termed pyogenic granuloma, is a benign vascular tumor. The gingiva, lips, tongue, and buccal mucosa are the most common sites of mucosal LCH and it rarely involves nasal cavity. Anterior nasal septum has been reported as most common origin in nose (1, 2).

The etiology of LCH remains unknown but trauma and hormonal changes, such as those that occur in pregnancy, are thought to be major etiologic factors. Viral oncogenes, microscopic arteriovenous malformations, cytogenetic abnormalities and production of angiogenic factors are also associated with LCH (1). In four cases that nasal packing is mentioned as a causative factor, the sites of origin of lesion were inferior and middle turbinates as well as sphenopalatine region (6-9). 

A giant pyogenic granuloma of the posterior part of the nasal septum that occurred after prolonged use (30 days) of a nasogastric feeding tube has also been recorded (10). In a study of 40 patients with LCH over a period of 20 years, 6 patients (15%) had nasal trauma and 2 patients (5%) had pregnancy as risk factors (11). According to patient history, both factors (pregnancy and trauma) are presumed to be major etiologic factors in our case.

 Clinical symptoms include nasal obstruction, Epistaxis, Epiphora, purulant or mucous rhinorrhea and nasal deformity (4). In a study on 40 patients, unilateral epistaxis and nasal obstruction occurred in 95% and 35% of patients respectively (11). In our case, although she was completely asymptomatic during her pregnancy, nasal obstruction and mild episodes of epistaxes started about one month postpartum and gradually became aggravated over the following 10 months. 

The treatment of choice for these lesions is complete excision using endoscopic approach even for large lesions. In our case, there was no recurrence after complete endoscopic resection during one year follow up.
